# Case Report: Liver as a Source of Hematopoietic Stem Cells After Liver Transplantation Following Hematopoietic Stem Cell Transplantation

**DOI:** 10.3389/fped.2022.861692

**Published:** 2022-03-23

**Authors:** Tomasz Jarmoliński, Monika Rosa, Blanka Rybka, Renata Ryczan-Krawczyk, Kornelia Gajek, Katarzyna Bogunia-Kubik, Maja Klaudel-Dreszler, Piotr Czubkowski, Piotr Kaliciński, Joanna Teisseyre, Marek Stefanowicz, Ewa Gorczyńska, Krzysztof Kałwak, Marek Ussowicz

**Affiliations:** ^1^Department and Clinic of Pediatric Oncology, Hematology and Bone Marrow Transplantation, Wrocław Medical University, Wrocław, Poland; ^2^Laboratory of Clinical Immunogenetics and Pharmacogenetics, Laboratory of Tissue Immunology of the Medical Center, Hirszfeld Institute of Immunology and Experimental Therapy, Polish Academy of Sciences, Wrocław, Poland; ^3^Department of Gastroenterology, Hepatology, Nutritional Disorders and Pediatrics, Children’s Memorial Health Institute, Warsaw, Poland; ^4^Department of Pediatric Surgery and Organ Transplantation, Children’s Memorial Health Institute, Warsaw, Poland

**Keywords:** Fanconi anemia, liver transplantation, pediatric, hematopoiesis, graft-versus-host disease (GVHD)

## Abstract

We report a child with Fanconi anemia who, after hematopoietic stem cell transplantation (HSCT) complicated by acute graft-versus-host disease (GVHD), underwent orthotopic liver transplantation (OLT). Approximately 1 month after OLT, the presence of third-party genetic material from the liver donor was noted and in the next few weeks, the chimerism assessment revealed 100% liver donor leukocytes in the peripheral blood. The rapidly progressing GVHD with gut involvement resulted in patient’s death 6 months after OLT. The liver can act as a clinically significant source of hematopoietic stem cells, and the liver donor’s young age must be emphasized as potentially predisposing to this phenomenon. Transfer of OLT hematopoietic stem cells may not have clinical significance unless the patient is not immunocompetent or develops liver-transplantation associated GVHD, that can result in lymphocyte mediated elimination of original hematopoiesis. Patients with preexisting immunity disorder (such as primary or secondary immunodeficiency) might require intensified immunosuppressive therapy in peritransplant period as a prevention of liver-transplantation associated GVHD. Close monitoring of hematopoietic chimerism after OLT is warranted in patients at risk, because cytopenia or OLT hematopoiesis can reflect subclinical GVHD and further studies are necessary to elucidate this phenomenon.

## Introduction

In patients with end-stage liver failure due to hepatic graft-versus-host disease (GVHD) after hematopoietic stem cell transplantation (HSCT), orthotopic liver transplantation (OLT) is the therapy of choice. OLT for this indication is very rare, and since the first attempts, only approximately one hundred cases have been described in adults and children (1–4). Among possible OLT complications, an incidence of liver transplant-associated GVHD was described, but only one such case was reported among patients with OLT transplanted after HSCT (5). Here, we report a patient who, after HSCT complicated by acute GVHD resulting in vanishing bile duct syndrome and progressing to chronic liver insufficiency, underwent OLT due to irreversible liver injury. She was the first HSCT and OLT recipient presenting fatal liver transplantation-associated GVHD, and replacement of bone-marrow donor hematopoiesis by liver donor hematopoiesis was confirmed by chimerism studies.

## Materials and Methods

At the time of the study, mononuclear cell (MNC) and T-lymphocyte chimerism was evaluated. The MNC isolation procedure employed from blood or bone marrow was a Ficoll-Hypaque gradient. Ficoll-Hypaque solution has a specific gravity of 1.077 at room temperature and is denser than lymphocytes, and monocytes, but less dense than granulocytes and RBCs, allowing for the successful separation of these cell populations. T-lymphocytes were enriched by positive selection using a magnetic cell separation technique with CD3 magnetic beads and MiniMACS separator (Miltenyi Biotec, Bergisch Gladbach, Germany) according to manufacturer instruction. All results of posttransplant chimerism studies were based on short tandem repeat fragment analysis of variable number short nucleotide repeats using the AmpFlSTR SGM PLUS amplification kit (Thermo Fisher Scientific, Warrington, United Kingdom) with a sensitivity of 1%.

## Case Description

A 13-year-old girl with Fanconi anemia was referred for allogeneic HSCT from 10/10 HLA-matched unrelated donors due to bone marrow failure. The patient was prepared according to the modified GEFA03 protocol: busulfan 4 × 0.5 mg/kg b.w., fludarabine 6 × 30 mg/m^2^, cyclophosphamide 2 × 20 mg/kg b.w. and anti-thymocyte globulin (ATG; Grafalon, Neovii, Rapperswil, Switzerland) 3 × 20 mg/kg b.w., and GVHD prophylaxis consisted of cyclosporin A (CsA) and mycophenolate mofetil (MMF). The posttransplant course was uneventful with typical but mild complications (such as neutropenic fever, mucositis, and BKV replication). Successful engraftment with complete donor chimerism was observed on posttransplant day +12. The patient was discharged home on day +28 but was readmitted 3 days later with BK virus-related hemorrhagic cystitis and cutaneous manifestation of microangiopathy. Antiviral treatment with cidofovir was administered, and CsA was stopped. The girl developed grade III acute GVHD with skin, gut and liver involvement and was treated with corticosteroids and MMF. Despite a good response in the skin and gut, liver injury was observed, with elevation of aminotransferases (ALT – 1877 U/l, AST – 689 U/l) and gamma-glutamyl transpeptidase activity (GGTP, 2521 U/l) and jaundice with a maximum total bilirubin concentration of 29.7 mg/dl.

Multimodal immunosuppressive treatment with etanercept, basiliximab, ruxolitinib, plasmapheresis and extracorporeal photopheresis (ECP) was administered, but laboratory results showed that liver failure had progressed. Liver biopsy revealed vanishing bile duct syndrome – a typical histological picture related to end-stage liver GVHD. In view of the biopsy findings and Model of End-Stage Liver Disease (MELD) score of 19 points, the patient was placed on the liver transplant list. Eleven months later and 16 months after HSCT, she underwent a liver transplant from a deceased donor. Shortly after OLT, CMV replication was observed, and the patient was treated with ganciclovir, foscarnet and human cytomegalovirus immunoglobulin. Approximately 1 month after OLT, trilineage cytopenia with severe neutropenia not responding to filgrastim was noted. Bone marrow (BM) biopsy revealed aplasia with erythrophagocytosis, and secondary hemophagocytic syndrome related to CMV infection was initially suspected. The patient was transferred to the stem cell transplantation unit for hematological treatment. At that time, decreased bone marrow donor chimerism was recorded, and the patient was treated for 3 days with ATG, at a cumulative dose of 60 mg/kg b.w.

In hematopoietic chimerism studies, the presence of third-party genetic material was noted, and using frozen liver donor lymph node samples, the new genetic profile was identified to be compatible with the liver donor material. HLA typing confirmed complete HLA incompatibility between the patient (HLA-A*02:01/A*32:01, HLA-B*18:01/B*44:03, HLA-C*04:01/C*05:01, HLA-DRB1*03:01/DRB1*11:01, HLA-DQB1*02:01/DQB1*03:01) and the liver donor (A*24.33; B*38.51; DRB1*13.15).

**FIGURE 1:**
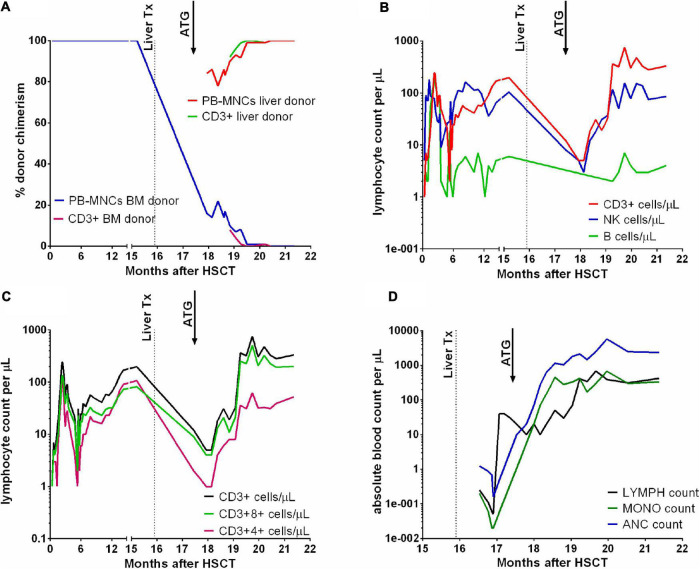
Chimerism results in peripheral blood mononuclear cells (PB-MNCs) and T lymphocytes **(A)**. T, B lymphocyte and NK cell counts **(B)**, and T CD3+4+ and CD3+8+ counts **(C)** after HSCT and OLT. Absolute lymphocyte (LYMPH), monocyte (MONO) and neutrophil (ANC) counts in the peripheral blood **(D)**. Dotted lines mark the time of liver transplantation, and arrows indicate the time of ATG therapy.

The first chimerism measurement after ATG therapy revealed 78% liver donor MNCs in the peripheral blood, and the percentage increased up to 100% in the next few weeks. Bone marrow chimerism revealed 36% HSCT donor cells and 64% OLT donor cells and single chimerism assessment in the nucleated cells showed 84% OLT donor cells (Figure 1).

Due to a continuous decrease in HSCT donor chimerism and progressive hematopoietic failure, 3 months after OLT, CD34-enriched peripheral blood stem cells from the same BM donor at a total of 5.3 × 10^6^ cells/kg b.w. were infused without conditioning as a stem cell boost but without any effect. One week later, the patient experienced severe abdominal pain with vomiting and bloody diarrhea, hyperbilirubinemia and elevated GGTP activity. Treatment with oral budesonide capsules, intravenous methylprednisolone at a dose of 2 mg/kg b.w. and etanercept was administered, but due to rapid clinical progression, basiliximab and ECP therapy were added. Endoscopic examinations revealed diffuse esophagitis, erosive gastritis and duodenitis with histological confirmation of acute GVHD. The rapidly progressing signs of GVHD with gastrointestinal tract functional obstruction led to the performance of surgical gastrojejunal anastomosis with the Roux-Y loop, which caused partial but transient improvement. As a consequence of fulminant GVHD progression with multiorgan failure, the patient died 21 months after HSCT.

## Discussion

Liver transplantation is feasible in end-stage liver disease HSCT survivors, but is associated with worse outcome than in general population. According to the multicenter survey conducted by Burdick et al. in patients after hematopoietic stem cell transplantation in childhood, the overall survival rate at 1 and 5 years after OLT was 74 and 63.2%, respectively (6). According to the study by Brockmann, best results of solid organ transplantations (SOT) after HSCT are achieved, if the SOT is carried up no sooner than 2 years after HSCT, from a living donor and with minimization of immunosuppressive treatment (3).

Graft-versus-host disease induced by liver transplantation is regarded as a severe and often fatal event occurring in 0.1–2.0% of OLT recipients, and is associated with a very high mortality rate, reaching 35% in children and 85% in adults (7, 8). Since the first report by Burdick et al. the pathogenesis was explained by the presence of a significant number of liver donor lymphocytes transferred with the organ in a number equivalent to the load in a bone marrow transplant (up to 10^9^–10^10^ cells) (6). Liver transplantation-associated GVHD yields symptoms similar to those of transfusion-associated GVHD, with skin and gut involvement and bone marrow aplasia, but the liver may remain unaffected. Risk factors associated with an increased incidence of liver donor-mediated GVHD are recipient age greater than 50 years, donor-recipient age difference of more than 20 years, younger donor age, HLA class I mismatch and glucose intolerance (9).

The unusual clinical course in the reported patient was associated not only with liver donor GVHD but also with expansion of liver donor hematopoiesis, revealing an unknown phenomenon in which the mature liver can act as an organ containing hematopoietic stem cells. To fully understand the meaning of reported chimerism values it must be noted, that the MNC product of Ficoll-Hypaque isolation from healthy individuals contains on average 30% monocytes and 60–70% lymphocytes (10). Lymphocytes originate from common lymphoid progenitor, and monocytes are descendants of common myeloid progenitor – the same as granulocytes, megakaryocytes and erythroblasts/erythrocytes (11). In the reported patient, observation of 100% OLT chimerism in MNC fraction at multiple timepoints was an evidence of full OLT myeloid chimerism, due to the fact that monocytes and lymphocytes represent separate lines of hematopoiesis.

It can be hypothesized that both transfer and durable engraftment of OLT donor hematopoiesis were caused by a combination of a few uncommon factors. First, the transplant recipient was in a state of profound immunosuppression, which helped the passenger lymphocytes eliminate the recipient’s hematopoiesis and prevented the recipient’s immune system from eliminating the liver-derived hematopoietic stem cells. Second, the liver was collected from a 10-year-old donor, which might have had a physiologically higher ability to shield and transfer hematopoietic stem cells. The liver is the most important blood-producing organ in fetal life, with hematopoietic activity expiring at birth, except for rare pathological conditions, such as osteopetrosis or osteomyelofibrosis. Nevertheless, it is probable that the liver environment can act as a temporary niche harboring hematopoietic stem cells even without the ability to provide a functional hematopoietic site. There are scanty and historical data regarding the presence of such cells in an adult liver and of their reactivation after liver transplantation (12, 13). Only one report documented such a finding in a 9-year-old girl who underwent OLT from a 12-year-old donor but did not develop GVHD (14). In contrast to the case presented by Alexander et al. we observed rapid engraftment of liver donor hematopoiesis with severe and fatal GVHD. Both of these cases share young ages of both the donors and the recipients, which can be responsible for physiological differences compared with the adult population.

## Conclusion

This case unravels the fact that the liver can act as a clinically significant source of hematopoietic stem cells, and the liver donor’s age factor must be emphasized as potentially predisposing to the expansion of liver donor hematopoiesis. Transfer of OLT hematopoietic stem cells may not have clinical significance unless the patient is not immunocompetent or develops liver-transplantation associated GVHD, that can result in lymphocyte mediated elimination of original hematopoiesis. Patients with preexisting immunity disorder (such as primary or secondary immunodeficiency) might require intensified immunosuppressive therapy in peritransplant period as a prevention of liver-transplantation associated GVHD. This stands in opposition to the recommendation on minimizing immunosuppression maintenance, suggested by Brockmann et al. but intensive immunosuppression should be used until the risk of transplantation associated GVHD subsides (3). According to study by Murali et al. median time to GVHD onset from liver transplantation was 28 days (interquartile range, 21–38 days) (8). Liver transplantation recipients must be surveilled on individual basis for hematological complications for at least 4–6 weeks. Close monitoring of hematopoietic chimerism after OLT is warranted in patients at risk, because cytopenia or OLT hematopoiesis can reflect subclinical GVHD and further studies are necessary to elucidate this phenomenon.

## Data Availability Statement

The datasets for this article are not publicly available due to concerns regarding participant/patient anonymity. Requests to access the datasets should be directed to the corresponding author.

## Author Contributions

TJ and MR: data collection, analysis, and manuscript preparation and acceptance. BR, RR-K, KG, and KB-K: data collection, molecular studies, and manuscript acceptance. MK-D, PC, PK, JT, MS, EG, and KK: data collection, patient care, and manuscript acceptance. MU: concept, analysis, and final acceptance. All authors contributed to the article and approved the submitted version.

## Conflict of Interest

The authors declare that the research was conducted in the absence of any commercial or financial relationships that could be construed as a potential conflict of interest.

## Publisher’s Note

All claims expressed in this article are solely those of the authors and do not necessarily represent those of their affiliated organizations, or those of the publisher, the editors and the reviewers. Any product that may be evaluated in this article, or claim that may be made by its manufacturer, is not guaranteed or endorsed by the publisher.

## References

[1] Rhodes DF Lee WM Wingard JR Pavy MD Santos GW Shaw BW Orthotopic liver transplantation for graft-versus-host disease following bone marrow transplantation. *Gastroenterology*. (1990) 99:536–8. 10.1016/0016-5085(90)91039-9 2365200

[2] Teisseyre M Teisseyre J Kalicinski P Wolska-Kusnierz B Ismail H Bernatowska E Liver transplantation for severe hepatic graft-versus-host disease in two children after hematopoietic stem cell transplantation. *Transplant Proc*. (2010) 42:4608–10. 10.1016/j.transproceed.2010.09.170 21168746

[3] Brockmann JG Broering DC Raza SM Rasheed W Hashmi SK Chaudhri N Solid organ transplantation following allogeneic haematopoietic cell transplantation: experience from a referral organ transplantation center and systematic review of literature. *Bone Marrow Transplant*. (2019) 54:190–203. 10.1038/s41409-018-0255-9 30082851 PMC7092162

[4] Faraci M Bertaina A Dalissier A Ifversen M Schulz A Gennery A Solid organ transplantation after hematopoietic stem cell transplantation in childhood: a multicentric retrospective survey. *Am J Transplant*. (2019) 19:1798–805. 10.1111/ajt.15240 30586230

[5] Schlitt HJ Tischler HJ Ringe B Raddatz G Maschek H Dietrich H Allogeneic liver transplantation for hepatic veno-occlusive disease after bone marrow transplantation–clinical and immunological considerations. *Bone Marrow Transplant*. (1995) 16:473–8. 8535323

[6] Burdick JF Vogelsang GB Smith WJ Farmer ER Bias WB Kaufmann SH Severe graft-versus-host disease in a liver-transplant recipient. *N Engl J Med*. (1988) 318:689–91. 10.1056/NEJM198803173181107 3278235

[7] Akbulut S Yilmaz M Yilmaz S. Graft-versus-host disease after liver transplantation: a comprehensive literature review. *World J Gastroenterol*. (2012) 18:5240–8. 10.3748/wjg.v18.i37.5240 23066319 PMC3468857

[8] Murali AR Chandra S Stewart Z Blazar BR Farooq U Ince MN Graft versus host disease after liver transplantation in adults. *Transplantation*. (2016) 100:2661–70. 10.1097/TP.0000000000001406 27495762 PMC5118135

[9] Elfeki MA Pungpapong S Genco PV Nakhleh RE Nguyen JH Harnois DM. Graft-versus-host disease after orthotopic liver transplantation: multivariate analysis of risk factors. *Clin Transplant*. (2015) 29:1063–6. 10.1111/ctr.12627 26358521

[10] Vissers MCM Jester SA Fantone JC. Rapid purification of human peripheral blood monocytes by centrifugation through Ficoll-Hypaque and Sepracell-MN. *J Immunol Methods*. (1988) 110:203–7. 10.1016/0022-1759(88)90104-4 2837516

[11] De Kleer I Willems F Lambrecht B Goriely S. Ontogeny of myeloid cells. *Front Immunol*. (2014) 5:423. 10.3389/fimmu.2014.00423 25232355 PMC4153297

[12] Wang XQ Lo CM Chen L Cheung CKY Yang ZF Chen YX Hematopoietic chimerism in liver transplantation patients and hematopoietic stem/progenitor cells in adult human liver. *Hepatology*. (2012) 56(4):1557–66. 10.1002/hep.25820 22544823

[13] Uzoka C Tzvetanov IG Patel P Benedetti E. Pancytopenia due to graft versus host disease post liver transplantation: a case report. *Hematol Med Oncol*. (2018) 4:1–4. 10.15761/HMO.1000176

[14] Alexander SI Smith N Hu M Verran D Shun A Dorney S Chimerism and tolerance in a recipient of a deceased-donor liver transplant. *N Engl J Med*. (2008) 358:369–74. 10.1056/NEJMoa0707255 18216357

